# Using Simulation Exercises to Deal With the Death of a Child as Part of Healthcare Studies

**DOI:** 10.7759/cureus.53284

**Published:** 2024-01-31

**Authors:** Lasse Tervajärvi, Sanna Tervajärvi, Stiina Storvik-Sydänmaa, Nina Hutri

**Affiliations:** 1 Education, Tampere Centre for Skills Training and Simulation, Tampere University of Applied Sciences, Tampere, FIN; 2 Education, Virrat Comprehensive School, Virrat, FIN; 3 Pediatric Nursing, Tampere University of Applied Sciences, Tampere, FIN; 4 Pediatrics, Faculty of Medicine and Health Technology, Tampere Centre for Skills Training and Simulation, Tampere, FIN

**Keywords:** teaching by simulation, death of a child, family in crisis, teaching and training medical and nursing students and faculty, interprofessional education and collaboration

## Abstract

Background: Almost all healthcare professionals find themselves in a situation where they witness death in their work. Meeting a family in crisis is challenging for healthcare professionals and students. Simulation is an effective tool to practice complex and emotionally challenging situations in healthcare education.

Methods: The aims of the study were to find out what challenges healthcare students experience when facing a family in crisis and to assess the usability of simulation in teaching healthcare students how to manage this situation. Voluntary simulations for paramedic students and medical students (a total of 29 students) were held in the autumn of 2021. Before and after the simulations, the students evaluated their skills to meet a family in crisis (the loss of a child) with the help of a questionnaire that contained mostly open-ended questions. The study was completed using a qualitative method.

Results: The challenges raised by our students were divided into three categories: child-, family-, and self/student-related. Child-related challenges included the developmental stage which impacted communication. Family-related challenges included family members’ different reactions to a devastating situation. Student/self-related challenges were few previous child contacts, communication with the family, and a situation that requires paying attention to multiple things at the same time.

Conclusion: According to our study, the students find simulation as a useful method for preparing to communicate with a family in crisis. The students see that with the help of simulation, they can practice different modes of operation and communication when facing a family in crisis. They also have the possibility to think about the processing of their own emotions during a crisis.

## Introduction

In the Western world, the death of a child is a rare event that few healthcare students go through during their clinical practical training [1]. It has impact on both the family and the medical staff [2]. Despite the fact that witnessing death is inevitable in health care, little research has been done on simulations dealing with the death of children in particular [3]. End-of-Life-Care (EOLC) simulation is a relatively new way of preparing the students for practical work [4]. With the help of the simulation, students are able to experience the emotionally challenging and complex situation that comes with the death of a child. Without teachers’ experience, it is difficult to teach students how to take care of a child and her/his family or to appreciate different feelings and thoughts when seeing a child’s death [5].

Simulations about a family dealing with death underline the importance of planning and the quality of debriefing discussion [6]. Even in simulation, death is an emotional event; hence without proper debriefing, simulation can leave students with negative feelings such as shame and inadequacy [6].

## Materials and methods

The aim of the study was to find out what challenges the students experience when meeting a critically ill child and a family in crisis and what kind of things they think should be taken into account in that kind of situation. In addition, we wanted to hear how useful the students find these simulations in terms of the development of their professional skills. We received permission to conduct this study from Tampere University.

The simulations were part of an interprofessional course between paramedic students and medical students. The paramedic students were third-grade students (3/4 years) and the medical students were in the clinical phase (5-6/6 years). 29 students participated in the voluntary simulations organized in August 2021. The students were divided into three groups that participated in two simulations. There were about 10 students at a time in the simulations. The simulations dealt with meeting a family in crisis and the sudden unexpected death of a child. In the first simulation, the topic was the severe intoxication of an adolescent, and in the second, the sudden unexpected death of an infant. In the simulation of sudden infant death syndrome (SIDS), the role of the relative was played by a teacher and simulation facilitator with acting experience. The decision to stop CPR was made by the pediatrician who was also one of the trainers.

Beforehand, the students knew that the simulations would deal with meeting a family in crisis and they would have a pediatrician who would help them to go through the difficult situations. Debriefing was after each case and it was goal-oriented. Each debriefing lasted 50-60 min and it was held by three teachers specialized in pediatrics. During the debriefing, we discussed the treatment of the child, causes of the situation, how to meet a family in crisis, and also a lot about how children differ from adults and why many healthcare personnel find it hard to treat children. In the sudden unexpected death of a child, it was made clear that there was no way to save the patient. We teachers also told the students how we have met families in crisis and handled our own feelings. Students were allowed and encouraged to ask any questions concerning the matter.

The students assessed their own abilities to encounter a family in crisis and the death of a child by filling in questionnaires. The questionnaires were answered via email both before and after the simulations. Twenty-three (79 %) participants answered the initial survey and 11 (38 %) participants answered the final survey. Answering the questionnaires was voluntary, collected data was anonymous, and therefore, our research was ethically acceptable. If the answering had taken place during the simulation, the response rate would probably have been bigger.

In the initial survey, students were asked, for example, to tell about their own readiness for meeting with a family in crisis, their own experiences with a crisis situation, and what they think is difficult about facing an acutely ill child and family in crisis. In the final survey, the students were asked, among other things, what they think is important when dealing with a family in crisis and whether such a simulation was useful. In addition, they were asked once again what is the most difficult thing about facing a family in crisis.

The study was carried out as qualitative research. In the analysis of the data, data-driven content analysis has been used, which aims to create categories or concepts that describe the phenomenon and build a conceptual system or model from them [7].

Since the number of respondents to the survey was small, we did not separately analyze the answers of the initial and final surveys but went through them together. We started analyzing the material by carefully studying the answers we received, after which we derived an initial set of themes of the answers around our research questions. After deriving the themes, we started to reduce the data. From the reduced expressions, we collected subcategories, which we summarized into main categories. For example, our first research question dealt with difficulties the students meet. This became the theme “difficulties the students face” for which we started to collect answers. The answers were summarized and divided so that we were able to create three different categories. These were the factors related to a dying child, a family, and the student her/himself. Finally, we clarified under these categories the things the students had linked to them, for example to the factors related to a child the students mentioned the stages of a child’s development, individuality, and the size of a child compared with an adult.

## Results

The preliminary questionnaire assessed how the death of a child has been discussed during studies and what the students find challenging when encountering an acutely ill child and a family in crisis.

**Figure 1:**
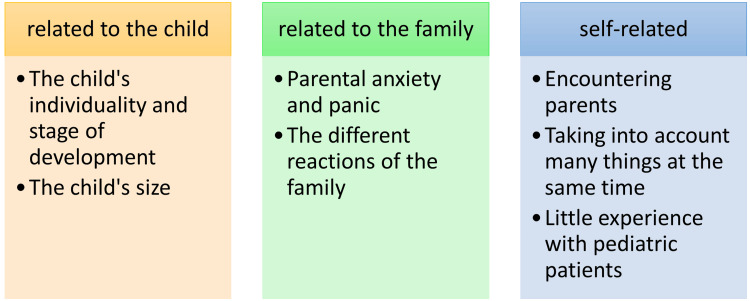
According to the students, factors that complicate the encounter between an acutely ill child and her/his family.

Encountering an acutely ill child and her/his family is always difficult. When analyzing the students’ answers, the difficulties were divided into three topics, which were related to the child, the family, or the person working as a healthcare professional (Figure 1).

The factors related to the child deal with the child's individuality and stage of development when the child is a patient. The students point out that they don't necessarily know what a child's normal level of development is, and what one can expect from the child. In addition, children develop individually at different stages, for example in terms of speech. The healthcare personnel do not know the child's individual stage of development, and for instance, the reaction to strangers may vary. The child may not be able to verbalize what she/he feels. Furthermore, the child is small compared to an adult, which can make procedures and dosing of medicines difficult. Gamble et al. [8] point out that pediatrics is its own special field, in which case the special characteristics of children and adolescents should be highlighted in all functions during education.

“*The individuality of the child, for example in terms of developmental level and others. What is normal for a child and what isn’t?*”

Family-related factors include parental anxiety and panic and the family’s various reactions. The students mention how accepting parents’ different emotions can feel difficult and finding the right words impossible.

“*Caring for parents’ distress, because the parents can be very worried (understandably) about their child and their “inability” to help their child is quite a distressing experience for them. In this case, the patient’s relatives are also often angry, and even if it is not directed directly at the treating entity, it is easy to let it get under the skin.*”

Out of the self-related factors, the most recurring one was meeting parents. Students point out how difficult it is to receive and accept parental anxiety. Communication with parents can also cause difficulties, as giving incorrect information or finding the right way to be present can seem difficult. Many students (n=17) mention that paying attention to both the child patient and parents at the same time poses challenges; suddenly it is not only the patient to be treated but also her/his family and family’s distress. The last of the self-related factors is the limited experience with pediatric patients. Most of the students regard their own experience with children as too little.

“*That what should be said or whether one should say anything. That if I say something “stupid” is it the only thing that the family will remember.*”

In the questionnaire after the simulations, it was assessed which thing the students saw as important when encountering a family in crisis and how they experienced the simulations.

**Figure 2:**
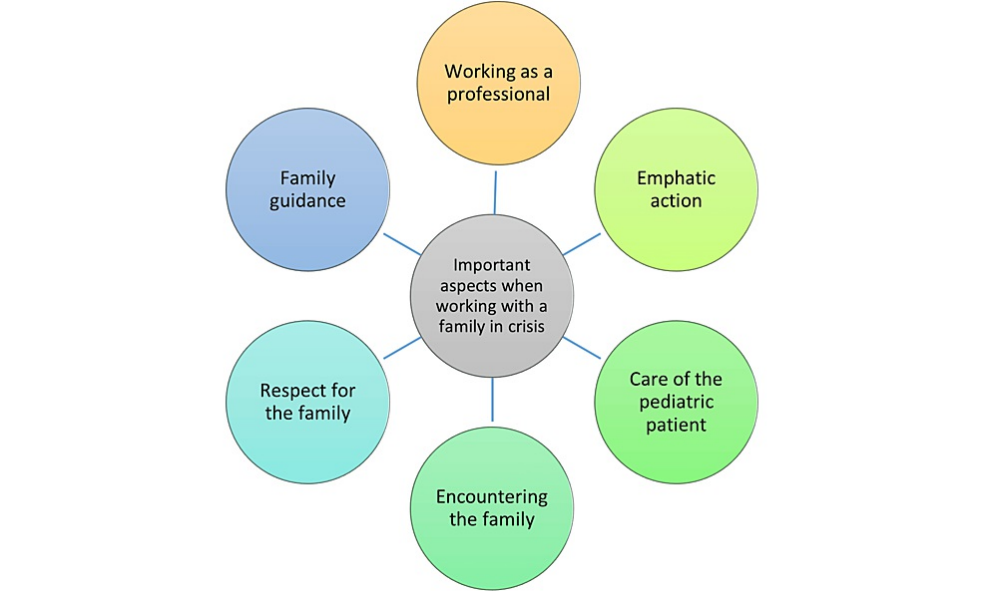
Important aspects highlighted by the students when working with a family in crisis.

The important issues mentioned by the students in their encounter with a family in crisis are summarized in Figure 2. Working as a professional means having sufficient theoretical knowledge, managing practical skills, cooperating with different actors, and managing one’s own emotions. In addition to empathy, empathic behavior includes gestures, listening, and being present. The care of pediatric patients includes knowledge of the child’s special characteristics, reaction to them, and interaction with the child. Encountering the family includes interacting with everyone involved in the situation and listening and accepting the parents’ concerns. Respect for the family, on the other hand, means recognizing the parents’ position as their child’s special expert and enabling the family’s activities. In this study, cultural aspects did not come up, unlike, for example, in the study by Cole and Foito [5]. In family guidance, in turn, the emphasis is on giving adequate information and direct guidance to the family to get the help and support they need.

All students who answered the final questionnaire felt that the training was useful for their future profession. Based on the final questionnaire, the training was especially praised for the fact that the families’ diverse reactions were addressed in a comprehensive manner and concrete advice was also available. Although, according to the students, it is not possible to fully prepare for crisis situations with the help of theoretical knowledge and simulation, they felt that the training was beneficial. The students emphasized that the simulation helps to create routines for their own way of acting and reduces tension in the future when the real situation comes.

Based on the training, development ideas were also presented. One of these was the availability of staff. The students pointed out that the staff was always available during the exercises, although in reality the situation may be different. Furthermore, some of the students hoped that the education would include the handling of practical arrangements after the death of a child from a perspective other than healthcare so that they would be able to answer the families’ questions if necessary.

## Discussion

As already stated earlier, the death of a child is a rare situation in the Western world. Due to its rarity, it is particularly important that healthcare professionals practice working and coping in these situations during their studies. Students see mentally demanding simulations of death as useful and positive learning experiences [4]. Heller et al. [9] state that, if used in the right way and responsibly, even death can be used in simulation teaching.

In their answers, the students pointed out that the death of a child has not been discussed sufficiently during education, which is also evident from, for example, the study by Weiss et al. [10]. In addition, the students mentioned that they do not feel that they have enough experience in encountering a family in crisis or a child’s death. Many students have limited experience with children, so interaction, communication, and developmental assessment can seem challenging [8].

When preparing for simulations on death, good planning is paramount. The instructor must have sufficient knowledge of simulations to be able to lead the simulation and conduct a satisfactory debriefing discussion afterward. Getting the right actors for the roles is also important. In simulation handling the death of a child, those who play the roles of the relatives must have some experience or training in acting [11], as we had.

Lindsay criticizes discussing the death of a child only through traditional teaching methods, such as lectures [12]. She mentions that they provide students with content, but they don’t really promote critical thinking skills or managing one’s own emotions. Therefore, we tried to provide students with the opportunity to face death during the simulation, so that teaching about death is not just theoretical. The students felt that the simulations helped them build routines and reduced tension. In several studies [4,13], simulations covering death have indeed been found to reduce students’ anxiety. The simulation also gives the chance to practice working and communicating specifically with children, even though the mannequin can never react completely like a child [8].

Grabow underlines that simulation dealing with death gives an opportunity to focus more on the learner instead of the patient [14]. The simulation is a safe learning environment where there is room to make mistakes and practice one’s skills, for example in terms of communication [4]. As in others [e.g. 10], this study also showed an increase in students’ self-efficacy related to seeing death.

As our students did not have much previous experience of witnessing the death of a child, the simulations were seen as particularly beneficial. Students commented that during the simulation, they received concrete advice on how to act or what to say when encountering a family in crisis or the death of a child was beneficial. For example, Pawley et al. note that the treatment received by family can have a great impact on how they cope with their grief [15]. Additionally, the simulations allowed our students to face the different reactions of family members. Seeing the various reactions gives the students the opportunity to work on their own emotions in difficult situations. For instance, Grabow reminds us to include a psychological perspective in simulations [14].

## Conclusions

The loss of a child is a great loss for parents, but it is also a difficult and challenging situation for healthcare professionals. The simulations dealing with the encounter of a family in crisis and the death of a child give a unique opportunity to practice the situation in a safe environment. According to our study, the students find simulation as a good method to practice facing a sick child and a family in crisis. The students see that with the help of simulation, they can practice different modes of operation when facing a family in crisis and have the possibility to think about the processing of their own emotions during a crisis. In the final questionnaire, we found out that despite the demanding and emotional nature of the subject, the students stated that these simulations were helpful. Future studies on the simulation of bereavement for medical students are warranted.
